# A parent motivational interviewing program for dental care in children of a rural population

**DOI:** 10.4317/jced.51662

**Published:** 2014-12-01

**Authors:** Mauricio González-Del-Castillo-McGrath, Juan-Manuel Guizar-Mendoza, Catalina Madrigal-Orozco, Laura Anguiano-Flores, Norma Amador-Licona

**Affiliations:** 1 Universidad De La Salle Bajío, Facultad de Odontología, León Guanajuato, México; 2 UMAE HE 1 Bajío, Instituto Mexicano del Seguro, Facultad de Odontología, León Guanajuato, México

## Abstract

Objectives: To determine the effectiveness of a motivational interviewing-based educational program in reducing the number and intensity of new caries and bacterial dental plaque levels at 6 months post randomization. Study Design: A randomized and single blind clinical trial in 100 schoolchildren between 6-10 years of age presenting the highest risk score of caries according to the Caries Management by Risk Assessment (CAMBRA) criteria was performed. These patients were randomized to two groups: control (in which the mothers initially received an oral prevention informative session) and experimental (in which the mothers received the initial informative session, followed by individual motivational interviewing sessions during a period of 6 months). The International Caries Detection and Assessment System (ICDAS) scores and bacterial plaque were evaluated at baseline, at 6 and 12 months. Results: After 12 months, children in the experimental group had 2.12 ± 0.8 new caries versus 3.5 ± 0.9 in the control group (t=7.39; p<0.001). Caries in the experimental group was seen to be limited to the enamel, with a median intensity of 2 (range 0-3) versus 3 (0-6) in the control group (U=1594; p<0.0001). Bacterial plaque determined by the O’Leary index decreased in both groups; however, it decreased more in the experimental than in the control group (34.3 vs. 20.6; t=-3.12, p= 0.002) respectively. Conclusions: Motivational interviewing is better than traditional educational programs in preventing caries and decreasing bacterial plaque.

** Key words:**Health educational, motivational interviewing, caries risk.

## Introduction

Dental caries is highly prevalent throughout the world, and affects between 60-90% of the school population (1,2). On the other hand, it is now possible to prevent or control most oral diseases or disorders in schoolchildren. As an example, coronal caries have decreased among individuals between 5-17 years of age in the United States (3). However, this has not happened in our country, where recently Guido *et al.*, reported caries prevalence from 94.7% to 100% in children residents of rural Mexico (4).

The most widely held idea of caries is that of a cavity, and only when the latter appears or begins to cause pain do parents seek help for their affected children (5-7).

The tendency unfortunately is to avoid treatment until it can no longer be postponed (7). On the other hand, restoration limits the damage caused but is unable to stop the physiopathological process of caries (6).

Since the 1960s, dental practice has increasingly focused on prevention. Educational methods for the promotion of health have been evaluated (8-10), mostly in the form of traditional or unidirectional programs based on informative sessions imparted by health professionals, leaflets, or public media campaigns (9). This type of approach has not been effective in modifying habits or in reducing the prevalence of caries (8,10), since the knowledge gained is only effective over the short term and is unable to modify behaviors (7).

Motivational interviewing [MI] is a psychopedagogic tool developed in recent decades with the primary objective of providing patients with intrinsic motivation to achieve and retain behavioral changes (11). It has been successfully used in establishing preventive behaviors related to diseases such as diabetes or HIV infection / AIDS (12,13). Weinstein *et al.*, used this type of tool for the first time in application to preventive behaviors among the mothers of infants at a high risk of developing caries from an early age (14-16). MI is grounded on the establishment of dialogue and confidence between the patient and the instructing health professional. Conversation is guided to allow patients to define behavioral changes on their own accord, with a view to obtaining personal benefit. Once the patients are able to identify personal motivation for change, the instructor provides the information needed to achieve such change, based on a range of strategies to be selected by the patients and incorporated to their daily life, in accordance with the capacity of each concrete individual.

This study compares parental information plus motivational sessions versus only parental information in reducing the number and intensity of new caries and bacterial dental plaque levels in children from a rural population.

## Material and Methods

A randomized and single blind clinical trial was carried out in the primary school “Praxedis Guerrero”, in the community of San Juan de Otates, Leon, Mexico. The inclusion criteria were children both genders, between 6-10 years of age and with the highest risk of developing caries according to the Caries Management by Risk Assessment [CAMBRA] criteria (17). Exclusion criteria were those cases in which the parent or caretaker failed to attend two or more motivational sessions, as were those lacking dental re-evaluation.

- Sample size: The sample size was calculated in order to provide 80% power to detect a difference of 35% in new caries between groups at a significance level of 5%. The resulting number of subjects was 45 children per group and it was decided to study 50 considering a 10% rate of losses during follow-up.

We selected 100 schoolchildren meeting the inclusion criteria, whose parents accepted their participation. They were randomly assigned to either of two groups [parental information plus follow-up motivational sessions or only parental information]. The selected children and their parents were informed about the purpose of the study, and written informed consent was obtained in all cases. The protocol was evaluated and approved by the research ethics committee of the University, in full accordance with ethical principles, including the World Medical Association Declaration of Helsinki [version 2008].

- Procedures: The primary outcome measure was dental caries at baseline, six and twelve months post randomization. A secondary measure was the plaque score at the same time.

Children´s teeth were evaluated using the International Caries Detection and Assessment System [ICDAS] score (18,19), which classifies caries progression from its incipient stages to manifest tooth destruction on permanent and primary teeth. A new caries was defined when it appeared in a tooth without decay in a subsequent review. The O’Leary score was used to assess bacterial dental plaque. A single investigator evaluated all children ignoring their group assigned, who was trained with the plaque [O’Leary score] and caries criteria [ICDAS score]. Intra-examiner agreement was high for both criteria [Kappa 0.83 and 0.88, respectively].

The mothers of all children included in the study received a 45-minute audiovisual informative session on the etiology and consequences of caries as well as methods for preventing the disease such as correct tooth brushing techniques. Mothers in the control group only received this session and subsequently their children were evaluated and treated for caries at six and twelve months later, reinforcing the preventing information of the disease.

Mothers in the experimental group received 6 periodic individual motivational interviewing counseling in addition to the oral health informative session. All sessions were conducted by two counselor members of the study team previously trained for this purpose and were certified if their MI skills. The counselor attempted to establish a therapeutic alliance, identify and reinforce dental needs. Children in this group were also treated for caries as the control group.

During the second session in the interviewing group we applied the Readiness Assessment of Parents concerning Infant Dental Decay [RAPIDD] scale (20), which identifies three stages of resistance to change: pre-contemplative, contemplative and action. This session lasted about 45 minutes and was divided into three previously protocolized phases. The first phase [Approach] was used to establish confidence between the instructor and the mothers of the children through dialogue involving open questions referred to the general family scenario. Specifically, questions of the following kind were asked: How many children do you have? How does your child behave? What does your child like to do? This was followed by questions designed to identify perceptions referred to dental health. In this phase the instructor helped create awareness of the problem in order to define a basis for actions aiming to produce change. In the second phase [Motivations for change]; the wishes of the mothers in relation to the dental health of their children were determined, with identification of the personal motivations requiring reinforcement: If you were granted a wish for the teeth of your child, what would it be? What do you expect in future for your child’s teeth? On a scale of 1 to 10, how much do you wish your child not to have dental problems? The fathers in turn formulated postulates for change: I want my child to have healthy teeth because…”, which were constantly underscored. Finally, the third phase [Initiatives for change] was used to discuss the options and strategies for satisfying the motivations for change and for improving dental health. In this context, the parents defined their preferences, adjusted to their capacity. The purpose of this phase was to develop a written plan for change. In addition, the parents received a number of playing cards referred to the selected strategies, and a calendar with vacant boxes. The parents were instructed to tick each box as confirmation of compliance with the strategies, or to leave the box empty on those days when the dental health strategies were not followed.

A reinforcement visit was programmed after two weeks and then every three weeks until 7 subsequent sessions were completed. On each occasion the motivations sustaining the changes were reinforced, with the identification of problems and the adoption of solutions.

Six and twelve months after randomization, both study groups were again evaluated by means of the ICDAS and O’Leary plaque scores.

Children in the two groups were interrogated about food intake between meals and soft drink consumption in a typical week.

## Statistical Analysis

Quantitative data are reported as mean and standard deviation, while qualitative variables are presented as percentages. The Student t-test for independent groups was used to compare groups, and repeated-measures analysis [ANOVA] was used to contrast initial, intermediate and final changes by dental plaque control and caries. The Mann-Whitney U-test was used for comparing new caries between the groups. Statistical significance was considered for *p*<0.05. The analysis was done with the package SPSS v. 21.

## Results

We studied 100 patients [50 in each group]. Four subjects were excluded during follow-up in the study: three in the control group [they did not accept final evaluation] and one in the experimental group [parents showed less than 80% adherence to motivational sessions and did not participate in the final evaluation]. The final study analysis thus involved 47 children in the control group [21 males and 26 females] and 49 in the experimental group [29 males and 20 females]. Mean adherence to the intervention program was 90%.

There were no differences in food intake between meals, 1.55 vs. 1.61 [Z=0.39, *p*=0.69] or soft drink consumption 1.42 vs. 1.51 [Z=0.69, *p*=0.48] for control and experimental group respectively.

No difference was found in the number of caries between groups; however, experimental group showed lower plaque control than the control group at baseline ( Table 1).

**Table 1:**
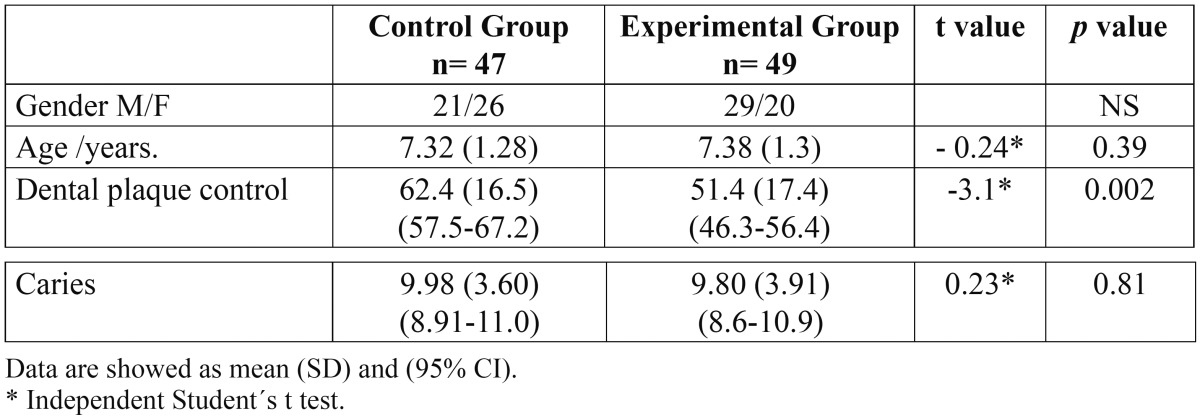
Baseline demographic data, dental plaque control and caries evaluation in rural children.

Dental plaque control and caries decreased in both groups during the study ( Table 2). However, dental plaque decreased more in the experimental than in the control group [34.3 vs. 20.6; t=-3.12, *p*= 0.002].

**Table 2:**
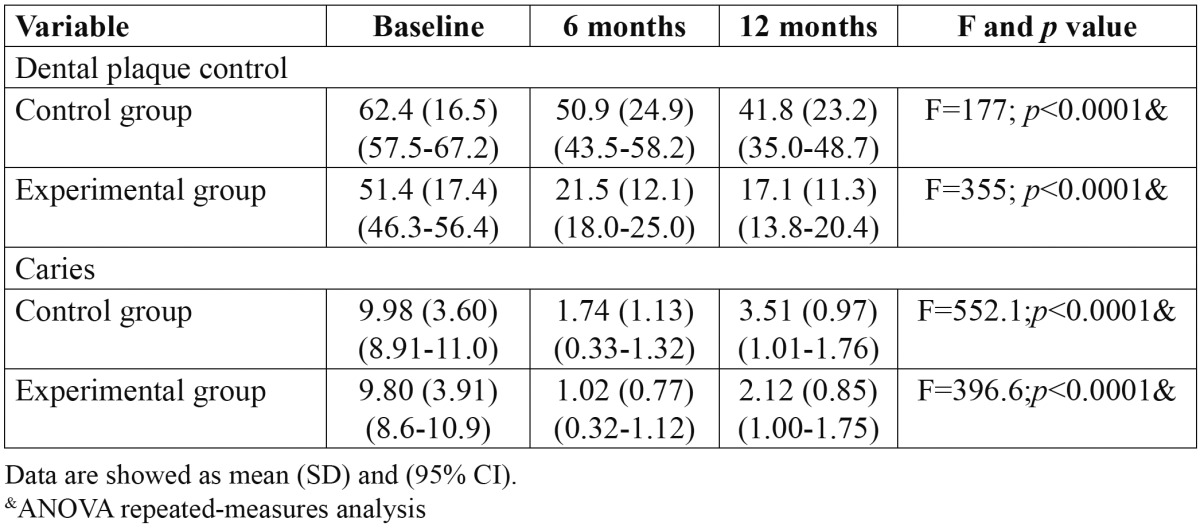
Dental plaque control and caries evaluation between groups in rural children.

The number of new caries was lower in the experimental than in the control group at the end of the study [2.12 vs. 3.51; t=7.39, *p*<0.0001] respectively. Also the intensity of caries according to the ICDAS criteria was lower in the experimental [2 [0-3]] than in the control group [3 [0-6]]; *p*<0.0001, at the final evaluation.

Regarding the initial stage of resistance to change based on the RAPIDD instrument, most mothers were in the contemplative stage [n=29, 59.2%], while 7 were in the pre-contemplative stage [14.3%] and 13 in the action stage [26.5%]. At the end everyone was in the action stage.

The postulates or reasons motivating change in the experimental group were: “To prevent my child from suffering pain” [n=16, 37%]; “For health reasons” [n=16, 37%]; “For aesthetic reasons” [n=4, 8.2%]; and “To prevent my child from losing teeth” [n=5, 10%]. No reason was given in four cases [8%].

Regarding the offered strategies, the most widely selected options were tooth brushing after each meal, the avoidance or reduction of sugars, sweets and fried foods to once a day, and the providing of water for school, instead of soft drinks. The options showing the best compliance were the provision of a tooth brush for personal use, the providing of water for school, instead of soft drinks, and tooth brushing after each meal ( Table 3).

**Table 3:**
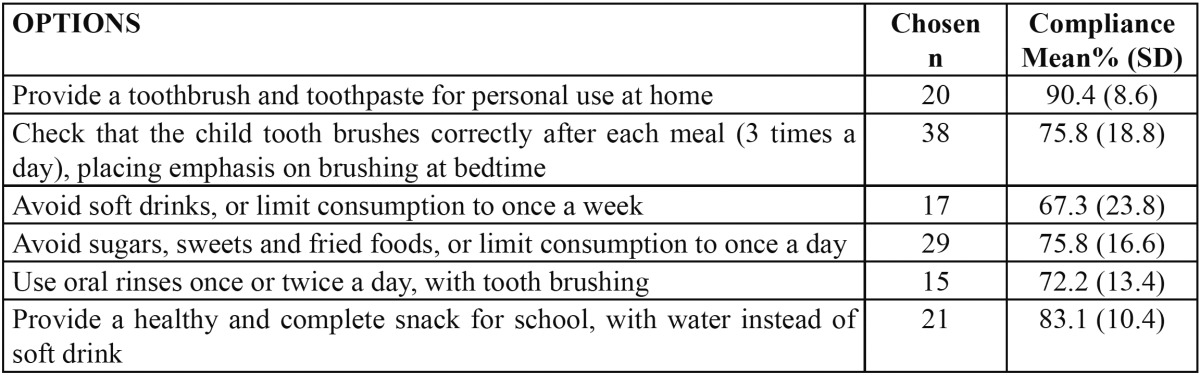
Frequency of options chosen by parents and those showing the best compliance in the experimental group.

## Discussion

The results of the present study indicate 37% fewer new caries detection and lower plaque score in rural children after a parent motivational interviewing program for dental care than in the control group with only an informative session on the etiology and consequences of caries. Although the time to re-evaluation was short, this is an acceptable period for the physiopathological progression of caries, allowing reliable clinical measurement of the lesions (21,22). Weinstein *et al.* found that the differences between the motivational interviewing group and the controls become firmly established over time (14). After two years, and in the absence of any new interventions, the same author found a lesser incidence of caries in the experimental group [35% versus 52%] than in the control group (15). This may reflect reinforcement of adherence to the preventive strategies as familiarization with a given habit increases.

Initially, and according to the questionnaire based on the RAPIDD scale, most of the mothers [59.2%] were in a contemplative stage as regards change. This implies the existence of important ambivalence: although the problems caused by bad habits are recognized, there is reluctance to change them. This may be attributed to a wish to avoid being bothered by such problems, to fear of failure, or a lack of interest (19). However, as the mothers attended the successive reinforcement sessions, compliance with each of the health strategies was seen to increase.

In recent years, the health systems of different countries have replaced the use of traditional caries evaluation indexes – particularly the DMFT [Decayed, Missing and Filled Teeth] score – with the ICDAS index (18,19). The reason possibly may be that the ICDAS allows clinical evaluation of the caries process, from the appearance of a simple white stain to manifest destruction of the dental tissues – thereby serving as a tool for the development of an interceptive management plan (22). One of the most promising findings in our study was that although the experimental group showed evidence of new caries, their progression in all cases was limited to the enamel [ICDAS grades 1-3], while in the control group they had progressed to manifest lesions, dentinal caries and tooth destruction in 29% of the cases [ICDAS grades 4-6].

The beneficial remineralization effects of fluor in caries has been widely studied (23,24). In the experimental group the final compliance rate was high in relation to tooth brushing after each meal as a strategy of change, in reference to the limitation of soft drink consumption, and in relation to the provision of a healthy and complete snack for school, with water instead of soft drink. These habits possibly favored oral microbiotic response to the development and progression of caries lesions, resulting in remineralization or containment of the disease. Both groups showed a tendency towards decreased bacterial plaque, particularly in the experimental group. Health promotion programs provide not only schoolchildren, but also their parents, with adequate information on dental care involving oral health habits and attitudes. The entire family should take responsibility for their dental hygiene (25). Although both groups received the traditional instructions referred to correct brushing technique and frequency, the group in which the parents were implicated in the brushing process showed the greatest plaque removal. Orienting mothers in health strategies, taking into account their primary needs, may strengthen initiatives to seek treatment both for themselves and for their children (26,27).

Some limitations of our study are: 1] we did not evaluate if these results persist over time or the impact of these educational techniques on other family members.

It was not the aim of our study to analyze the cost-effectiveness of the intervention, though such analyses could contribute to the future protocolization of health promoting initiatives of this kind. Adopting motivational interviewing strategies for the promotion of health could result in our country a clear reduction in the public costs generated each year as a result of restorative treatments, as has already been evidenced in California, where the triad education + CAMBRA + preventive treatment is regarded as a key element in the public oral healthcare system (28).

## Conclusions

Motivational interviewing is an effectiveness and promising technique for establishing preventive behaviors in relation to dental health, targeted to the parents of children at the highest risk for developing caries.
